# National Association of Dental Examiners

**Published:** 1887-09

**Authors:** 


### NATIONAL ASSOCIATION OF DENTAL EXAMINERS.

Reported for the Independent Practitioner.

The annual meeting of the National Association of Dental Examiners, a body made up of representatives from the State Boards appointed under the laws regulating dentistry, was held at the Park Theatre building, Niagara Falls, commencing Monday, August 1, 1887, Dr. J. Taft, of New York, being President, and Dr. F. A.

Levy, of New Jersey, Secretary. There were present at the opening the following members: From Illinois, Dr. G. H. Cushing; from Indiana, Dr. E. C. Kirk; from Ohio, Drs. H. A. Smith, J. Taft and C. R. Butler; from Kentucky, Dr. A. Wilkes Smith; from Massachusetts, Dr. L. D. Shepard; from New Jersey, Dr. Fred. A. Levy; from Maryland, Drs. E. P. Keech and T. F. Waters; from Alabama, Dr. C. P. Robinson, who stated, however, that he was not authorized to represent his board by any formal action.

The minutes of the previous meeting were read and approved. Dr. J. B. Willmott, of Toronto, a member of the Canadian board, was, upon motion, invited to the privileges of the floor. The chairman read a prepared order of business, which was adopted without debate.

The chairman first called for reports from the various boards, which were made by the representatives present, showing the number of applicants examined, the number of permits to practice granted, the manner of conducting examinations, and the status of the profession in regard to the State law. Comparatively few examinations were reported. Dr. Keech said that in Maryland every dentist, as far as they knew, was registered. Dr. Shepard said that Massachusetts had finally secured a law after repeated failures, and it had been obtained through the labor of a man who had not been identified with dental progress, nor with dental societies. Yet, at the sacrifice of considerable time and money, this dentist had obtained the passage of a law, when all the others had failed. The act was imperfect in some respects, every dentist who should hereafter enter upon practice being obliged to pass an examination, no matter from what college he was a graduate, the diploma of Harvard receiving no more recognition than those of the most distant school. There was one very singular fact in connection with the putting of the law into effect. During the last few days allowed for registration, more than fifty new signs were hung out in Boston, some being those of women, others of students, and some of children.

The methods of examination in the different States differed, in some it being entirely oral, while in others it was written, each examiner being allowed a definite number of questions, and required to mark the percentage of correct answers, the applicant falling below an established portion being rejected. In some boards no very systematic course was adopted, the examiners voting to pass or reject the candidate, according to the impression which he had produced upon their minds.

Dr. Kirk presented the cases of two colleges, concerning the diplomas of which the Indiana examiners desired the opinion of this Association. Graduates from the Louisville College of Dentistry, and from the dental department of the State University of Tennessee, had been refused permission to practice in Indiana. Graduates from the Louisville College of Dentistry had appeared before them, who could not name a single text-book that had been used in the college, nor was it possible to determine that the applicants had even really attended any full course of lectures. Students matriculated in March, 1887, were graduated in June of the same year. One of these showed no evidence of any knowledge of dentistry whatever.

The Tennessee College had repeatedly graduated students after attendance upon but one course of lectures.

Dr. Wilkes Smith, the Dean of the Louisville College of Dentistry, explained that the students complained of had been granted diplomas by the Dean of the Medical Department during the absence of Dr. Smith, and acknowledged that a mistake had been committed, which should not again occur.

Dr. W. C. Barrett, being present, was voted the privileges of the floor, and the chairman called upon him to express his views upon the subject at issue. He said that every member of this association should recollect that a grave responsibility rested upon him. In the competition for students, the colleges were liable to slacken the rules to admit applicants who should be rejected, and to promise graduation, in advance, to men who could not, in the limited time, qualify themselves. Villainous work had been done by colleges, and foreigners, especially, had found it easy to obtain diplomas. If the State Boards of Examiners stood up squarely to duty, they had it in their power to neutralize this, and to call the colleges to a strict accountability for the charge committed to them. But this was not to be done by letting down the standard in any individual instance, under the plea that an infraction of professional law was through a misunderstanding, and that although a diploma had been granted irregularly, it was due to inexperience or error. If a college assumed to grant diplomas, it was but fair to hold it responsible for the work done, and they should not be allowed to plead the baby act, but should be disciplined. Every school must be judged by its acts, and not by its intentions. If a mistake had been made by a school, let it manfully admit it, accept the proper punishment in a spirit of humility, and take care that no more such errors be chargeable to it.

The question of the Louisville Dental College was referred to a committee of three, to examine and report. Drs. Shepard, H. A. Smith and Keech were appointed such committee.

The question of the regularity of the dental department of the University of Tennessee was referred to a committee of three, consisting of Drs. Cushing, Butler and Keech.

At the Monday evening session Dr. Willmott appeared in behalf of the Royal College of Dental Surgeons, of Ontario. At a previous meeting the Board had declined to recommend its diplomas as sufficient qualification for practice. Against this he desired to enter a protest, and spoke for some time, urging a recognition of the Toronto diplomas. Upon motion this was referred to the same committee to which the Louisville College matter had been committed.

At the Tuesday afternoon session, this committee submitted the following report:

The committee to whom was referred the application of the Royal College of Dental Surgeons of Ontario, to have rescinded the action taken by this Association at its Minneapolis meeting, which decided that the L. D. S. should not be accepted as equivalent to a dental degree to save examination by the Board of Examiners, respectfully report that the action taken by this Association should stand, for the following reasons:

*First*—That the L. D. S. granted in Ontario is a local license to practice, not recognized in some of the Provinces of the Dominion, nor in Great Britain, rather than a degree in dentistry conferred upon the completion of a college education. In this respect it is analogous to the licenses granted by our State Boards, which are not generally recognized by the Boards of other States.

*Second*—That when L. D. S. is granted as a degree in dentistry, on the completion of the regular course in their school of dentistry, it represents two courses of four months each, while our rules require two courses of at least five months each.

*Third*—That in the last announcement they advertise to grant the L. D. S. for a fee, and after examination, upon any non-resident who has been in practice three years, exclusive of the two years of pupilage, *sine curriculo.*

Upon motion the report was unanimously adopted.

The second committee of reference reported as follows:

Your committee, to whom was referred the action of the Indiana State Board of Dental Examiners in relation to the rejection of the dental diplomas of the University of Tennessee, find that the University has granted dental diplomas to those who have attended but one course of lectures in any institution, which procedure is in direct opposition to the resolution of this Association adopted in 1884, in which it resolved that after the sessions of '84 and '85, the various State Boards composing the Association be instructed to refuse the diploma of any college which does not require as a prerequisite for graduation attendance upon two full, regular courses of lectures, and practical instruction of not less than five months' duration, and held in separate years.

The University of Tennessee must have been fully cognizant of this fact, as copies of the transactions of this association were forwarded to them, and the transactions were also published in the dental journals; hence they must have issued diplomas after the date above named, with the certainty that they would not be received by the State Boards represented in this body, and can therefore have no ground of complaint against any board for rejecting them.

Your committee find that the Indiana Board acted as in duty bound, and recommend that this Association fully endorse their action in this case.

Upon motion the report was unanimously adopted.

At the Wednesday afternoon session, the committee to whom was referred the Louisville Dental College matter reported as follows:

In a circular issued by the Secretary of the Indiana State Board of Dental Examiners, dated July 5, 1887, notice is given that "the Board has decided against accepting the testimonials from the Dental Department of the Hospital College of Louisville, Ky."

The Indiana Board has presented its statement of the case to this association, and the Dean of the Dental Department of the Central University of Kentucky (Louisville College of Dentistry) has given a full statement of its side of the case—has made a frank acknowledgment of the mistake made, and has given assurances that hereafter no one shall have cause to complain, and that so far as it is in the power of the college it will rectify this mistake and any others made.

Therefore, resolved: First—That the Medical Board only did its duty in refusing to accept diplomas from said college in this particular case.

Second—It is recommended to all State Boards to confer with a college whose diplomas are questioned before publishing it as derelict, to the end that no injustice may be done, particularly to those who have honestly earned their diplomas from such institution. It is possible that such a diploma might be issued without the slightest intention, on the part of the college, to depart from the standard governing the boards, as was claimed was the case in this instance.

The report was, on motion, unanimously adopted.

The Association elected as officers for the coming year:

*President*—Dr. Geo. H. Cushing, Illinois.

*Vice-President*—Dr. T. S. Waters, Maryland.

*Secretary*—Dr. Fred. A. Levy, New Jersey.

After the reading of the minutes, the Association adjourned to meet on the Monday preceding the next meeting of the American Dental Association.

